# Gender Disparities in Trauma Patients With an Elevated Shock Index at a Rural Appalachian Trauma Center

**DOI:** 10.7759/cureus.84347

**Published:** 2025-05-18

**Authors:** Aliya G Burns, Matthew Leonard, Hannah Collins, Bracken Burns

**Affiliations:** 1 Surgery, East Tennessee State University Quillen College of Medicine, Johnson City, USA; 2 Trauma, Ballad Health, Johnson City, USA

**Keywords:** gender disparities, rural, shock index, trauma, trauma outcomes

## Abstract

Introduction: Although men are more likely to experience a traumatic injury, research demonstrates that gender may impact patients’ biological responses to trauma. Shock index (SI) is a validated tool used to predict patient outcomes. This study aims to determine if gender is associated with differences in outcomes in patients with an SI ≥1.0 in the rural setting.

Methods: This retrospective study included 699 trauma patients with an SI ≥1.0 admitted to a rural trauma center from January 2017 to December 2022. Student’s t-test, chi-square analysis, and univariate logistic regression were used to analyze the study data. Primary outcomes included mortality and blood transfusions. Secondary outcomes included discharge to a care facility, hospital days, ICU days, and ventilator days.

Results: Males with a SI ≥1.0 had a significantly longer length of stay (LOS) (7.0 ± 7.4 vs. 5.8 ± 6.7, P = 0.039), had a higher injury severity score (14.5 ± 12.2 vs. 10.4 ± 10.1, P < 0.001), were x1.99 more likely to die (OR = 1.999, 95% CI = 0.213-1.171, P = 0.005), and were x1.7 more likely to require blood transfusion (OR = 1.700, 95% CI = 0.323-0.937, P = 0.002) than females. Males were 4.4 times more likely to have a motorcycle accident (OR = 4.4, 95% CI = 1.935-9.816, P < 0.001). Females were 1.5 times more likely to have a fall (OR = 1.5, 95% CI = 1.099-2.146, P = 0.012) and 1.6 times more likely to have an MVC (OR = 1.6, 95% CI = 1.141-2.175, P = 0.006).

Conclusion: These findings emphasize a gender disparity in trauma patients with an SI ≥1.0 at this study’s rural institution. This study adds to the literature on SI and gender disparities.

## Introduction

Gender disparities in trauma outcomes have been the subject of extensive investigation within the medical community. Studies have consistently shown differences in outcomes between males and females following traumatic events. Most interestingly, females tend to have increased survival rates after traumatic injuries [1,2]. Understanding these gender-based differences is crucial for optimizing patient care and developing targeted interventions to improve outcomes.

Shock index (SI), calculated by dividing heart rate by systolic blood pressure, is a validated tool used to predict patient outcomes. Allgöwer et al. first described SI in 1967 as a more effective way to identify impending circulatory collapse [3]. Patients with an SI >0.9 tend to present with increased rates of mortality and other adverse outcomes [4]. Previous research has shown outcome differences between males and females of an elevated SI in the urban setting, but no published studies to our knowledge examine these differences in the rural environment. In addition to mortality differences, these studies have noted longer hospital and ICU length of stay (LOS), higher blood transfusion rates, and longer ventilator days in males with elevated SI [4-6]. This study aims to determine if gender is associated with differences in outcomes in patients with an SI ≥1 and hypothesizes that gender disparities exist.

## Materials and methods

Study design

This study is an IRB-approved, retrospective, single-institution study.

Data collection

The study period was January 1, 2017, to December 31, 2022. Data were collected from the trauma registry of a rural level 1 trauma center.

Inclusion and exclusion criteria

Adult trauma patients with a calculated SI of ≥1 were included. Patients not arriving by emergency medical services (EMS) or with missing data points (most commonly prehospital vital signs in the early years of the study) were excluded from the dataset. Outliers of SI (defined as values greater than three) were excluded from the study. To help control the validity of LOS data, high outliers (patients with LOS greater than 38 days) were excluded. The length of 38 days was determined using a formula for LOS outliers. The formula utilized was third quartile LOS in days + 1.5(5) = 37.5.

Statistical analysis

SI was defined as the standard definition of heart rate divided by systolic blood pressure. Patients were separated into male and female cohorts. Student’s t-test was used to observe and compare average age, LOS, injury severity score (ISS), ICU days, and ventilator days between the genders. Chi-square analysis evaluated differences in mortality, discharge to a care facility, and blood transfusions between genders. Univariate logistic regression was used to evaluate differences in injury mechanisms between genders and to determine predictors of mortality and blood product requirements in males and females. Primary outcomes included mortality and blood transfusions. Secondary outcomes included discharge to a care facility (rehabilitation center or skilled nursing facility), hospital days, ICU days, and ventilator days. JASP (version 0.18.2; https://jasp-stats.org) was used for the analyses.

## Results

Six hundred ninety-nine patients aged≥18 had an SI ≥1, including 267 (38.2%) females and 432 (61.8%) males. Descriptive statistics showed that females and males presented at an average age of 40, which showed no significant difference between the genders. Males had significantly longer hospital stay days (7.0 ± 7.4 vs. 5.8 ± 6.7, P = 0.039) and higher ISS (14.5 ± 12.2 vs. 10.4 ± 10.1, P < 0.001) compared to females. Total ICU days and total ventilator days were not found to differ between the genders (Table 1).

**Table 1:** Student’s t-test evaluating the demographics and secondary outcomes between genders. SD = standard deviation; ISS = injury severity score

Variables	Male (Mean ± SD)	Female (Mean ± SD)	T-value	P-value
Age	40.5 ± 24.2	40.1 ± 27.7	-0.235	0.814
Total Hospital Days	7 ± 7.4	5.8 ± 6.7	-2.066	0.039
Total ICU Days	5.7 ± 5.3	5.2 ± 4.8	-0.837	0.403
Total Ventilator Days	4.9 ± 5.3	4.5 ± 4.8	-0.516	0.606
ISS	14.5 ± 12.2	10.4 ± 10.1	-4.552	< 0.001

Chi-square analysis revealed that males had a significantly higher mortality (n=78, 18.1%) compared to females (n=29, 10.9%; P = 0.010). A significantly higher proportion of males (n=177, 41.0%) required blood transfusion compared to females (n=79, 29.6%; P = 0.002) (Table 2).

**Table 2:** Chi-square analysis of mortality, discharge to care facility, and blood transfusion between males and females.

Variables	Male	Female	χ²	P-value
	Mortality			
Yes	78 (18.1%)	29 (10.9%)	6.588	0.010
	Discharge to Care Facility			
Yes	105 (29.7%)	77 (32.4%)	0.484	0.486
	Blood Transfusion			
Yes	177 (41.0%)	79 (29.6%)	9.214	0.002

**Figure 1:**
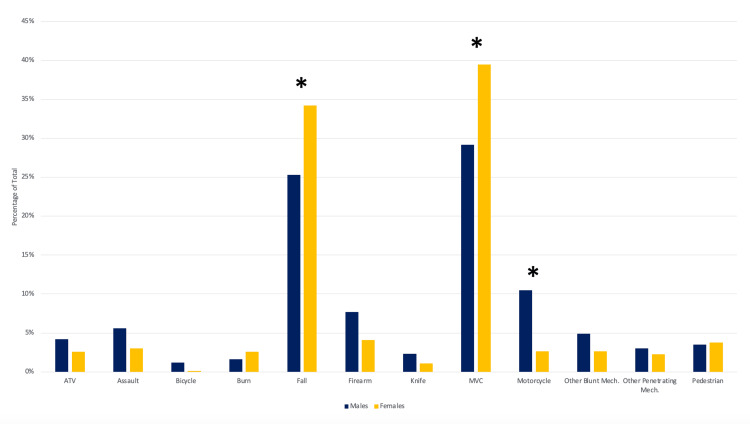
Differences in injury mechanism between males and females.

This study evaluated the following mechanisms of injury: ATV, assault, bicycle, biting, burn, fall, firearm, glass, knife, motor vehicle collision (MVC), machinery, motorcycle, other blunt mechanism, other penetrating mechanism, and pedestrian. Significantly, males were 4.4 times more likely to have a motorcycle accident (OR = 4.4, 95% CI = 1.935-9.816, P < 0.001). Also significantly, females were 1.5 times more likely to have a fall (OR = 1.5, 95% CI = 1.099-2.146, P = 0.012) and 1.6 times more likely to have an MVC (OR = 1.6, 95% CI = 1.141-2.175, P = 0.006). Additional mechanisms of injury were not found to differ significantly between genders (Figure 1).

Univariate logistic regression revealed that the odds of experiencing mortality for males were almost twice that of females (OR = 1.999, 95% CI = 0.213-1.171, P = 0.005). Although the p-value reflects significance, the confidence interval includes one, which suggests that the predictive mortality has no statistical significance. Regardless of gender, a one-year increase in age increased the odds of mortality by 3.2% (OR = 1.032, 95% CI = 0.023-0.041, P < 0.001). Males were 1.7 times more likely to require blood transfusion compared to females (OR = 1.700, 95% CI = 0.323-0.937, P = 0.002). Regardless of gender, a one-year increase in age increased the odds of blood transfusion by 1.9% (OR = 1.019, 95% CI = 0.013-0.025, P < 0.001) (Table 3). 

**Table 3:** Logistic regression of age and male gender on mortality and blood transfusion. CI = confidence interval

Variables	Odds Ratio	95% CI (Lower)	95% CI (Upper)	P-Value
Mortality				
Gender (Male)	1.999	0.213	1.171	0.005
Age	1.032	0.023	0.041	<0.001
Blood Transfusion				
Gender (Male)	1.700	0.323	0.937	0.002
Age	1.019	0.013	0.025	<0.001

## Discussion

SI was first defined in 1967 by Allgöwer et al. with the purpose of predicting impending circulatory collapse [3]. SI has since been extensively studied in the literature, especially within the context of traumatic injury, as a predictor of adverse patient outcomes. An SI of >0.9 has been linked to increased mortality and need for blood transfusion in the trauma patient population in both the urban and rural settings [4,5]. Other outcomes associated with an elevated SI include longer length of hospital and ICU stay and more ventilator days [6]. Given physiological changes that occur with aging and medications that alter blood pressure, McNab et al. studied the predictive value of SI among patients in different decades of life [6]. They determined that SI’s predictive value for poor outcomes holds up until the eighth decade of life [6].

Researchers have more recently been studying delta SI (ΔSI), a measure of the change in SI over time. It holds the potential to evaluate patient status more dynamically by suggesting either an improvement or decline in shock status. An elevated ΔSI has also been shown to have predictive value; it is notably linked with increased mortality and need for blood transfusions in the trauma patient population [7-10].

There has not been much research conducted on the SI and gender disparities. Trauma outcomes in this study revealed gender disparities among patients with an elevated SI. Male patients with an SI of ≥1 experienced significantly higher mortality and blood transfusion rates, longer hospital stays, and higher ISS rates compared to females with an SI of ≥1 at this rural level 1 trauma center. Logistic regression confirmed these associations, except for mortality, because the confidence interval crossed one.

In regard to the literature, there are few studies that evaluate the SI in terms of gender. Our study findings align with the literature suggesting that women have better outcomes than men after traumatic events. One study tried to elicit differences between male and female responses to trauma through measurements of serum lactate within 30 minutes of ED arrival and units of RBCs transfused. They found significantly lower lactate levels, RBC units transfused in the first 24 hours, and total units transfused in female patients after traumatic injury compared to males [11]. Notably, the female cohort in this study also had a significantly higher average ISS [11]. In our study, the higher ISS among our male patients may act as a confounding variable. In a future study, we might propensity score match our patients to confirm that women with elevated SI still have better outcomes.

Several retrospective analyses and prospective studies have contributed valuable insights into the survival patterns among trauma patients, shedding light on the impact of gender on mortality and physiological response to shock. Much of the literature suggests that hormones may play a role when it comes to protecting females against shock. Using the National Trauma Data Bank, one study noticed a significantly decreased mortality in female patients aged 13-64, which they categorized as hormonally active, after traumatic injury compared to males of the same age group [1]. Prepubertal (<13) and postmenopausal (≥65) women did not present with this protection against mortality [1]. Females in our study presented with this same protection, despite not controlling for age and hormone status. Future research might examine outcomes after better defining the hormone status of female patients, since the onset of puberty and menopause varies between women. Our study also revealed that differences between the genders with an elevated SI apply in the rural setting, which stands out against the many urban studies that have previously been conducted.

A study conducted on large-breed animals points to 17a-ethinyl estradiol-3-sulfate (EE-3-SO4) as the hormone providing women protection against shock [12]. Animals administered EE-3-SO4 immediately after an induced TBI and 40% blood volume removal exhibited a decreased mortality rate [12]. More research is needed to determine if EE-3-SO4 has the same effect in humans, but these findings might help explain what is being seen in clinical studies.

Limitations include those of a single-institution, retrospective study. Future research is needed to explore potential physiological mechanisms, especially the role of hormones, underlying these gender disparities. Despite not controlling for hormone status, this study still found that males presented with significantly worse outcomes. Subsequent studies should control for hormone status by separating women into categories such as premenopausal, perimenopausal, and postmenopausal and comparing their outcomes to those of men in similar stages of life. Of course, this may prove difficult due to women who have had an oophorectomy or are on hormone replacement therapy.

## Conclusions

Notably, our study demonstrates a lower mortality and blood transfusion rate among women with an elevated SI compared to men. In light of these findings, it is evident that gender plays a significant role in determining outcomes following traumatic events. In this study, this was shown to be true in patients with an SI ≥1 in the rural setting. Understanding the interplay between gender, physiological responses, and trauma outcomes is essential for advancing personalized medicine approaches and improving patient care in the field of trauma and critical care.
